# YOD1 regulates oxidative damage of dopamine neurons in Parkinson's disease by deubiquitinating PKM2

**DOI:** 10.1002/ctm2.70420

**Published:** 2025-07-18

**Authors:** Xia Zhao, Jinfeng Sun, Fan Chen, Hao Tang, Yuqing Zeng, Luyao Li, Qin Yu, Linjie Chen, Muzaffar Hammad, Xiaoxia Xu, Ziyao Meng, Wei Wang, Guang Liang

**Affiliations:** ^1^ Affiliated Yongkang First People's Hospital Hangzhou Medical College Yongkang China; ^2^ School of Pharmaceutical Sciences Hangzhou Medical College Hangzhou China; ^3^ Key Laboratory of Natural Medicines of the Changbai Mountain Ministry of Education Yanbian University Yanji China; ^4^ Chemical Biology Research Center School of Pharmaceutical Sciences Wenzhou Medical University Wenzhou China

**Keywords:** oxidative stress, Parkinson's disease, PKM2, YOD1

## Abstract

**Background:**

Parkinson's disease (PD) is a common neurodegenerative movement disorder, mainly characterized by the degeneration and loss of dopaminergic neurons in the substantia nigra. Oxidative stress is considered to be a key contributor to dopaminergic neuronal degeneration, triggering a series of downstream events such as mitochondrial dysfunction, neuroinflammation and misfolded protein aggregation, which ultimately exacerbate the development of PD. Deubiquitinating enzymes (DUBs) regulate oxidative stress, but their roles in PD remain unclear.

**Methods:**

GEO database analysis and western blotting were used to analyze the expression of YOD1in PD patients and PD mouse models. Genetic knockout (KO) of YOD1 was performed to assess its effects in PD pathogenesis. The substance of YOD1 was measured via co‐immunoprecipitation (Co‐IP) coupled with LC‐MS/MS analysis. Then the effect of YOD1‐mediated motor deficits and oxidative damage were investigated using open field test, swimming test, pole test, immunofluorescence (IF) and cellular analyses.

**Results:**

YOD1 was highly expressed in PD patients and 6‐OHDA‐induced PD model mice and mediated reactive oxygen species (ROS) production. YOD1 KO ameliorated motor impairments and oxidative stress in PD model mice. YOD1 directly bound PKM2 and reduces its ubiquitination level by removing the K63‐linked ubiquitin chain of PKM2, thereby increasing the tetramer level and reducing the dimer level of PKM2. It then inhibited dimerized PKM2 entry into the nucleus and regulated Nrf2‐mediated antioxidant responses, but YOD1 does not change the stability of PKM2 protein.

**Conclusions:**

Our study identifies YOD1 as a oxidative‐sensitive regulator of PD progression, operating via the YOD1‐PKM2‐Nrf2 axis. Targeting YOD1 may offer a novel therapeutic strategy for PD.

**Key points:**

- YOD1 is highly elevated in different PD model mice and patients with PD.
- YOD1 is a key regulator in oxidative stress and PD pathology.
- YOD1‐deficient exhibit a protective effect on neuronal oxidative injury.
- YOD1 targets PKM2-Nrf2 axis in response to oxidative stress.

## Graphical abstract

YOD1 binds to PKM2 and reduces its ubiquitination level by removing the K63 ubiquitin chain of PKM2, thereby increasing the tetramer level and reducing the dimer level of PKM2. It then inhibited dimerized PKM2 entry into the nucleus and activated the Nrf2 signaling pathway, thus promoting oxidative stress.

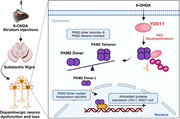

## 1 INTRODUCTION

Parkinson's disease (PD) is the second most prevalent neurodegenerative disorder after Alzheimer's disease, clinically characterized by cardinal motor symptoms including resting tremor, rigidity, bradykinesia and postural instability.^1^, ^2^ The global ageing population has driven a marked increase in PD prevalence, with current rates reaching 1.7% among individuals aged ≥65 years in China, projected to affect 5 million people by 2030.^3^ While the precise aetiology remains elusive, the pathological hallmark is the degeneration and loss of dopaminergic neurons in the substantia nigra. Current gold‐standard therapy relies on dopamine (DA) replacement strategies; however, long‐term treatment often leads to debilitating motor complications that substantially limit therapeutic efficacy. These clinical challenges underscore the critical need to elucidate the molecular mechanisms underlying dopaminergic neuron degeneration and identify novel therapeutic targets for more effective PD interventions.

Studies have shown that an imbalance between reactive oxygen species (ROS) production and elimination plays an important role in the pathogenesis of PD,^4^ as evidenced by increased ROS in the brains of PD patients.^5^, ^6^ Elevated ROS levels in PD brains directly contribute to dopaminergic neuron degeneration.^7^, ^8^ DA serves as a crucial neurotransmitter within the brain. The progressive degeneration and loss of dopaminergic neurons in individuals with PD result in diminished levels of DA within the striatum, thereby promoting the occurrence of disease. Notably, DA metabolism triggers compensatory increases in glutathione (GSH) levels, reflecting heightened oxidative stress. Under physiological conditions, endogenous antioxidant systems maintain redox homeostasis^9^; however, PD patients exhibit impaired antioxidant defences, rendering neurons particularly vulnerable to oxidative damage. The NF‐E2‐related factor 2 (Nrf2) signalling pathway serves as a key regulator of cellular antioxidant responses, regulating the transcription of protective enzymes including heme oxygenase‐1 (HO‐1) and NAD(P)H: quinone oxidoreductase 1 (NQO1).^10^ Disruption of Nrf2 signalling enhances susceptibility to oxidative insults, whereas its activation provides neuroprotection. Given these mechanistic insights, Nrf2‐targeted antioxidant therapies have emerged as a promising therapeutic strategy for PD.

Ubiquitination represents a dynamic, highly specific post‐translational modification that critically regulates diverse cellular processes, including protein degradation and apoptosis.^11^ Deubiquitinating enzymes (DUBs) counterbalance this process by selectively cleaving ubiquitin chains from target proteins, thereby modulating their stability and function.^12^ Recent studies have shown that DUBs play an important regulatory role in regulating oxidative stress. For example, USP7 inhibition attenuates ROS production and thus alleviates the damage caused by myocardial ischemia and reperfusion.^13^ Inhibition of USP15 mediates DNA damage in hematopoietic cells through the activation of NRF2 and intracellular ROS production.^14^ OTUD1 mediates KEAP1‐dependent antioxidant responses and oxeiptosis.^15^ And USP14 promotes ROS‐dependent ferroptosis in renal ischemia.^16^ Together, DUBs are involved in the regulation of oxidative stress in different diseases, but the key DUBs that regulate oxidative stress in PD and their mechanisms of action remain unclear. Our bioinformatic analysis of substantia nigra transcriptomes from PD patients versus controls revealed significant upregulation of YOD1, an OTU‐family deubiquitinase targeting K48‐ and K63‐linked polyubiquitin chains.^17^ Previous studies implicate YOD1 in mitochondrial stress responses through MAVS deubiquitination (K63‐specific)^18^, ^19^ and Huntington's disease pathogenesis via transcriptional regulation.^20^ Interestingly, we identified a novel role for YOD1 in potentiating oxidative stress and ROS generation in PD models, but the underlying mechanisms require further elucidation.

The study aimed to elucidate the key role and mechanism of YOD1 in mediating oxidative stress and PD pathogenesis. Our study demonstrated that YOD1 knockout attenuates oxidative damage in neuronal cells and ameliorates PD‐related pathology. Mechanistically, YOD1 regulates the redox‐sensitive PKM2 tetramer‐dimer equilibrium through K63‐linked deubiquitination. The subsequent nuclear translocation of PKM2 dimers potentiates Nrf2‐mediated antioxidant responses, ultimately mitigating oxidative stress. Together, this study demonstrates the potential of YOD1 as a novel therapeutic target for PD and provides a mechanistic foundation for developing selective inhibitors of the YOD1‐PKM2 signalling axis.

## 2 MATERIALS AND METHODS

### 2.1 General reagents

All chemical reagents and kits were obtained from commercial sources as follows: Dulbecco's modified Eagle's medium (DMEM; #D6429), dimethyl sulfoxide (DMSO; #D2650), bovine serum albumin (BSA, #10711454001; Sigma) and 6‐hydroxydopamine hydrobromide (6‐OHDA, #H4381) were bought from Sigma‐Aldrich. Cell culture supplements, including penicillin/streptomycin (#15140‐122) and Opti‐MEM (#31985070), were purchased from Gibco. Biochemical reagents included RIPA lysis buffer (#P0013B), MTT (#ST316), ROS assay kit (#S0033S) and JC‐1 assay kit (#C2006) from Beyotime Institute of Biotechnology. Apoptosis was assessed using an Annexin V‐FITC/PI detection kit (#556570; BD Biosciences). Western blotting supplies consisted of markers (#C520010; Sangon Biotech), PVDF membranes (#1620177; Bio‐Rad) and ECL substrate (#P10300; NCM Biotech). Antibody information is provided in Table S1, with primer and siRNA sequences detailed in Table S2.

### 2.2 Establishment of PD pathological model mice

Male C57BL/6J mice (wild‐type) and congenic YOD1 knockout (YOD1KO) mice were obtained from Shanghai Biomodel Organism Science & Technology Development Co., Ltd. Briefly, the YOD1 conditional knockout mouse model was generated using CRISPR‐Cas9‐mediated genome editing. Briefly, Cas9 mRNA and target‐specific gRNA were synthesized by in vitro transcription. A homologous recombination donor vector was constructed via In‐Fusion cloning, containing 3.0 kb 5′ and 3′ homology arms flanking a 1.3 kb loxP‐flanked (floxed) critical exon region. These components (Cas9 mRNA, gRNA and donor vector) were co‐microinjected into C57BL/6J fertilized zygotes to generate founder (F0) mice. Potential founders were screened by PCR amplification and Sanger sequencing of the target locus. Sequence‐verified F0 mice were subsequently bred with wild‐type C57BL/6J mice to establish the F1 generation. The male A53T α‐synuclein transgenic mice and age‐matched C57BL/6 wild‐type mice were obtained from GemPharmatech Co., Ltd. This well‐characterized PD model expresses human SNCA with the A53T mutation under the control of the mouse prion protein promoter, exhibiting progressive motor dysfunction and α‐synuclein pathology characteristic of PD. All animals were maintained under specific pathogen‐free conditions with ad libitum access to food and water in a controlled environment (22–25°C, 45–55% relative humidity, 12:12 h light‐dark cycle) at a density of five mice per cage. All experimental procedures were conducted in accordance with protocols approved by the Institutional Animal Care and Use Committee of Hangzhou Medical College (2023‐036).

#### 2.2.1 6‐OHDA‐induced acute PD models

YOD1KO mice and wild‐type (WT) mice (8 weeks, 22–24 g) were randomly divided into four groups (*n*  =  10/group): WT+sham, Model (6‐OHDA‐infused WT mice), YOD1KO+sham and YOD1KO+Model (6‐OHDA‐infused YOD1KO mice). For surgical procedures, mice were anesthetized with isoflurane (3% induction, 1.5% maintenance) and secured in a stereotaxic frame (Reward). An incision is made along the sagittal line of the head to expose the bregma. Place the microsyringe needle at the bregma and return the coordinates to zero. Injection coordinates of the striatum region were determined based on the Paxinos and Franklin atlas (−2.0 mm posterior to bregma, ± .5 mm lateral to midline, 2 mm deep from the dura). Using a skull drill (Reward) to make a small hole with a diameter of approximately 1 mm on the skull surface and inject drugs 2 mm below the skull (2 µL 6‐OHDA [5 µg/µL in 1% ascorbic acid/saline] for model groups, or 2 µL vehicle [1% ascorbic acid/saline] for sham controls) at a speed of.1 µL/min. After injection, leave the needle for 5 min and then slowly withdraw the needle.

#### 2.2.2 A53T chronic PD models

Male A53T mice and WT mice (6 months, 28–30 g) were randomly divided into three groups (*n*  =  10/group): WT+AAV‐CN, A53T+AAV‐CN and A53T+AAV‐shYOD1. Neuron‐specific YOD1 knockout (AAV‐shYOD1) AAV included: A neuron‐specific YOD1 knockdown vector: rAAV‐U6‐DIO‐shRNA(YOD1)‐3′CMV‐EGFP‐PA and A paired Cre recombinase vector: rAAV‐hSyn‐CRE‐WPRE‐hGH polyA. Viral particles were mixed at a 1:1 ratio and delivered via stereotaxic injection into the striatum under isoflurane anaesthesia.

### 2.3 Open field test

Prior to behavioural assessment, mice were acclimated to the testing room for 60 min to minimize stress‐induced variability. For open field testing (OFT), each mouse was gently placed in the centre of a square arena (40 cm × 40 cm × 40 cm) constructed of white polyvinyl chloride. Animal behaviour was recorded for 5 min using an overhead digital camera system connected to SuperMaze behavioural tracking software (Xinxin Technology). The system automatically quantified: total distance travelled (cm), average velocity (cm/s) and frequency of centre zone entries (defined as > 50% body length crossing into the central 20×20 cm area). Between trials, the apparatus was thoroughly cleaned with 75% ethanol to eliminate olfactory cues, and waste was promptly removed to prevent interference with subsequent tests.

### 2.4 Swimming test

Prior to testing, mice were acclimated to the experimental room for 60 min to minimize stress‐related behavioural variability. For swim testing, each mouse was gently placed in a circular water maze (diameter: 200 cm; water depth: 30 cm; maintained at 25 ± 1°C) under constant lighting conditions. Behavioural parameters were recorded for 5 min using an overhead video tracking system (SuperMaze v2.0, Xinyi Information Technology) to quantify: swim trajectory, total distance travelled (cm) and average velocity (cm/s).

### 2.5 Pole test

A custom‐designed wooden pole (1 cm diameter × 50 cm length) was constructed with a small cork ball of 2.5 cm in diameter fixed on the top. The pole surface was wrapped with medical‐grade gauze to enhance traction and secured at a 45° angle within the home cage environment. For testing, mice were initially positioned head‐down on the cork ball platform and allowed to descend voluntarily. The latency period was precisely measured from initial contact with the ball until both forepaws contacted the base platform using a digital stopwatch (resolution:.01 s). Each subject underwent three consecutive trials separated by 10‐min inter‐trial intervals in their home cage to minimize stress. The mean descent latency across trials served as the primary behavioural metric for motor coordination assessment.

### 2.6 Cell culture and transfection

PC12 and NIH3T3 cell lines were maintained in Dulbecco's Modified Eagle Medium (DMEM) supplemented with 10% foetal bovine serum and 1% penicillin/streptomycin (100 U/mL penicillin, 100 µg/mL streptomycin) at 37°C in a humidified 5% CO₂ incubator. Medium was replaced every 24–36 h, and cells were passaged at 80–90% confluence using.25% trypsin‐EDTA. For all experiments, cells in the logarithmic growth phase were utilized. For transfection experiments, cells were seeded in 6‐ or 12‐well plates at appropriate densities. Following the manufacturer's protocol (Lipofectamine 8000, #C0533FT, Beyotime Biotechnology), transfection complexes were prepared by separately mixing either plasmid DNA or siRNA with serum‐free DMEM in one sterile tube, and Lipofectamine 8000 reagent with serum‐free DMEM in another tube. After 5 min incubation at room temperature, the two solutions were combined, vortexed gently and incubated for an additional 15 min to allow complex formation. The DNA‐lipid complexes were then added dropwise to cells cultured in fresh complete medium. Transfected cells were maintained for 24 h prior to subsequent experiments.

### 2.7 Cell viability assay

PC12 cells were seeded in a 96‐well plate at a density of 1×10^4^ cells/well (100 µL complete medium/well) and allowed to culture overnight. Following experimental treatments, cells in each group were added with 10 µL MTT solution (5 mg/mL in PBS) and incubated for 3 h at 37°C. The formazan crystals formed were subsequently solubilized by adding 100 µL DMSO per well after careful removal of the supernatant. Plates were gently agitated for 10 min on an orbital shaker to ensure complete dissolution. Absorbance was measured at 490 nm using a microplate reader. Cell viability was calculated as: (OD experimental group/OD control group) × 100%.

### 2.8 Flow cytometry

Cell apoptosis was quantified using an Annexin V‐FITC/PI detection kit following the manufacturer's protocol. Briefly, treated PC12 cells were collected, washed three times with ice‐cold PBS and detached using.25% trypsin‐EDTA. The reaction was terminated by adding complete culture medium, followed by centrifugation at 1000 rpm for 5 min at 4°C. After supernatant removal, cells were resuspended in 190 µL of 1× binding buffer and stained with 5 µL Annexin V‐FITC and 5 µL propidium iodide (PI) for 30 min at 37°C in the dark. Apoptotic populations were immediately analysed by flow cytometry (BD FACSCanto II), with 10 000 events recorded per sample. Viable (Annexin V−/PI−), early apoptotic (Annexin V+/PI−), late apoptotic (Annexin V+/PI+) and necrotic (Annexin V−/PI+) cells were distinguished using FlowJo software (v10.8.1).

### 2.9 Co‐immunoprecipitation

Total protein was extracted using ice‐cold RIPA lysis buffer supplemented with protease and phosphatase inhibitor cocktails. Lysates were centrifuged at 12 000 rpm for 15 min at 4°C, with 50% of the supernatant retained as input control. For immunoprecipitation, the remaining supernatant was incubated with target‐specific primary antibody (1:1000) overnight at 4°C with constant agitation, using species‐matched non‐specific IgG as a negative control. Protein A/G magnetic beads were then added and incubated for 4 h at room temperature. Bead complexes were washed three times with 500 µL lysis buffer, and precipitated proteins were quantified by BCA assay. Samples were denatured in 2× Laemmli buffer at 95°C for 10 min prior to electrophoresis.

### 2.10 Western blot

Proteins were resolved on 10% SDS‐PAGE gels and transferred onto PVDF membranes at 260 mA for 90 min using a wet transfer system (Bio‐Rad). Membranes were blocked with 3% BSA in TBST for 1 h at room temperature, followed by overnight incubation at 4°C with primary antibodies diluted in blocking buffer. After three 10‐min TBST washes, membranes were incubated with HRP‐conjugated secondary antibodies for 2 h at room temperature. Following additional washes, protein bands were visualized using enhanced chemiluminescence substrate and imaged with a Bio‐Rad Gel Doc XR+ system equipped with Image Lab software.

Native PAGE gel, which preserves proteins' native conformation during separation, was used to detect PKM2 dimer and tetramer. Briefly, native PAGE gels were prepared by mixing the following components to achieve the desired acrylamide concentration: 30% acrylamide/bis‐acrylamide solution (29:1 ratio), 1.5 M Tris‐HCl (pH 8.8), 10% APS and TEMED. The separating gel solution was prepared first by combining appropriate volumes of acrylamide/bis solution, Tris‐HCl buffer and deionized water, followed by the addition of APS (.05%) and TEMED (.05%) to initiate polymerization. After pouring the separating gel, it was overlaid with isopropanol to ensure a flat interface. Once polymerized (typically within 30 min at room temperature), the stacking gel (4% acrylamide) was prepared using.5 M Tris‐HCl (pH 6.8) with the same initiator system. All gels were cast in 1.0 mm‐thick mini‐gel cassettes and allowed to polymerize completely before use.

### 2.11 Immunofluorescence

Following behavioural assessments, mice were perfused transcardially with ice‐cold PBS, followed by 4% paraformaldehyde (PFA). Brains were post‐fixed in 4% PFA overnight at 4°C, then cryoprotected in sequential 20% and 30% sucrose solutions (24 h each) until complete submersion. Tissue was embedded in optimal cutting temperature compound and sectioned coronally at 20 µm thickness using a CM3050S cryostat (Leica Microsystems). For immunofluorescence (IF), sections were permeabilized with.1% Triton X‐100 in PBS for 15 min at room temperature, followed by blocking with 10% BSA for 1 h. Primary antibodies diluted in blocking buffer were applied overnight at 4°C. After PBS washes, sections were incubated with Alexa Fluor‐conjugated secondary antibodies (1:500, Invitrogen) for 2 h at room temperature in the dark. Nuclei were counterstained with DAPI‐containing antifade mounting medium (Thermo Fisher), and images were acquired using a Nikon A1R HD25 confocal microscope with NIS‐Elements software.

### 2.12 LC‐MS/MS assay

NIH3T3 cells were transfected with either Flag‐YOD1 or empty Flag vector (Flag‐CN) control constructs using Lipofectamine 8000. After 24 h incubation, cells were harvested and lysed in RIPA buffer (50 mM Tris‐HCl pH 7.4, 150 mM NaCl, 1% NP‐40,.5% sodium deoxycholate) containing complete protease inhibitor cocktail (Roche). Flag‐tagged proteins were immunoprecipitated using anti‐Flag M2 magnetic beads (Sigma‐Aldrich) at 4°C for 4 h with rotation. Beads were washed three times with PBST (PBS +.1% Tween‐20), and bound proteins were eluted with 3×Flag peptide (100 µg/mL). Immunoprecipitated samples were resolved on 10% SDS‐PAGE gels and stained with Coomassie Brilliant Blue R‐250. Distinct protein bands from the Flag‐YOD1 and Flag‐CN groups were excised for in‐gel tryptic digestion. LC‐MS/MS analysis was performed by Jingjie PTM BioLab using a Q Exactive HF‐X mass spectrometer (Thermo Scientific) coupled with an EASY‐nLC 1200 system.

### 2.13 GEO database analysis

Gene expression datasets (GSE43490 and GSE7621) were retrieved from the Gene Expression Omnibus (GEO; https://www.ncbi.nlm.nih.gov/geo/), comprising postmortem substantia nigra transcriptomes from PD patients and age‐matched controls. Raw CEL files were processed using the robust multi‐array average normalization method in Affymetrix Expression Console (v1.4.1). YOD1 mRNA expression levels were extracted and compared between PD and control cohorts using Student's *t*‐test with Benjamini−Hochberg correction for multiple comparisons (FDR < .05). Differential expression was visualized using ggplot2 (v3.3.5) in R (v4.1.0), with significance thresholds set at |log2FC| > .5 and adjusted *p*‐value < .05.

### 2.14 Statistical analysis

All experimental data were collected and analysed under double‐blind conditions using GraphPad Prism 8.0 (GraphPad Software). Continuous variables are presented as mean ± standard error of the mean (SEM). Between‐group comparisons were performed as follows: Two‐group analyses employed unpaired two‐tailed Student's *t*‐tests; Multi‐group comparisons used one‐way ANOVA with Tukey's post hoc test (α = .05); and Factorial designs utilized two‐way ANOVA with Sidak's multiple comparisons correction. Statistical significance was defined as *p* < .05 for all analyses.

## 3 RESULTS

### 3.1 YOD1 is up‐regulated in PD models

**FIGURE 1:**
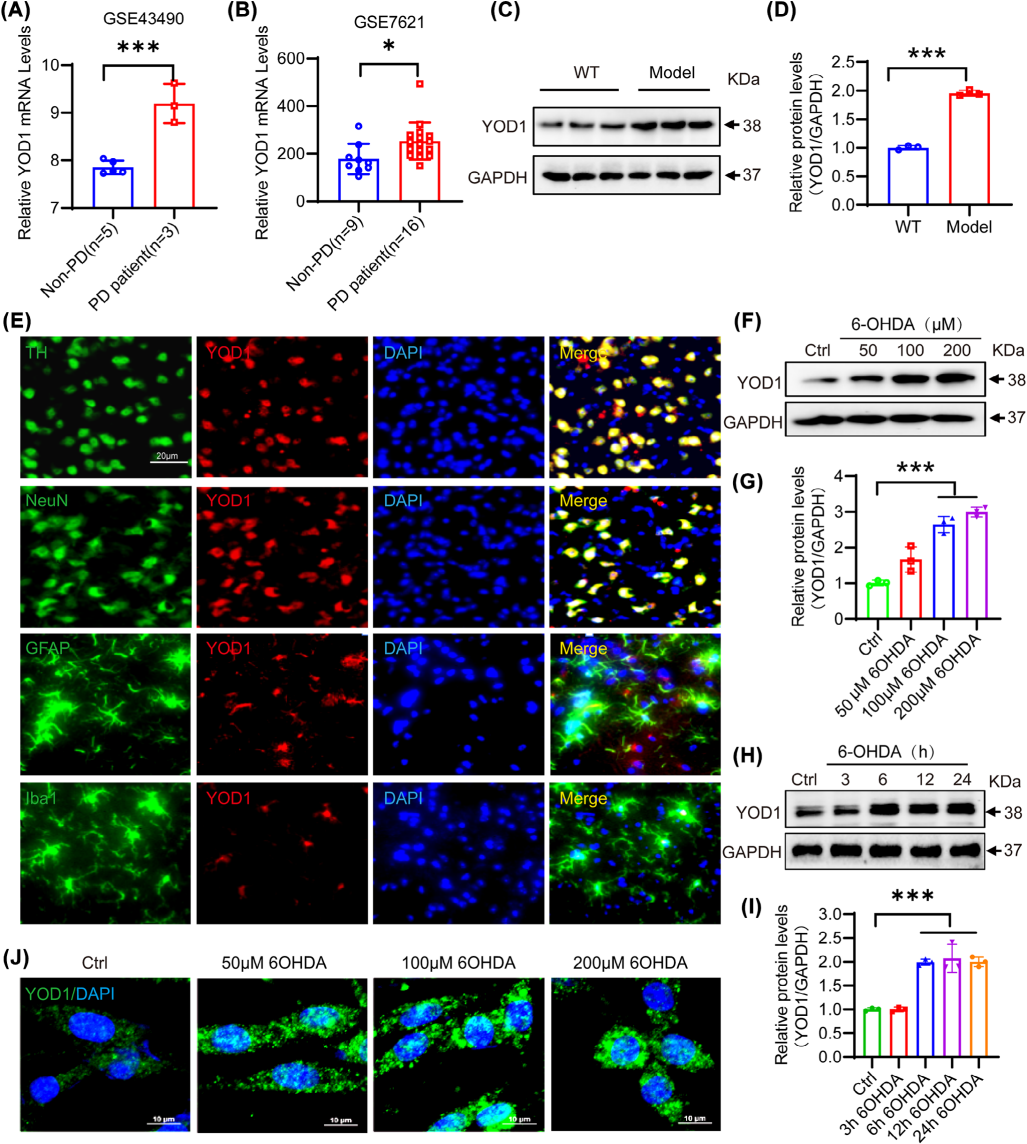
YOD1 is significantly elevated in PD animal models and patients with PD. (A, B) GEO database search showing relative YOD1 mRNA expression levels in the substantia nigra of PD patients and non‐PD patients. (C) Expression levels of YOD1 protein in the substantia nigra of C57BL/6 mice injected with 6‐OHDA (Model group) and saline (WT group) into the striatum. (D) Quantification analysis of YOD1 in C. (E) Immunofluorescence co‐staining shows YOD1 (red) expression in dopaminergic neurons (TH+), neurons (NeuN+), astrocytes (GFAP+) and microglia (IBA1+) (all green). (F) Expression level of YOD1 protein in PC12 cells after administration of gradient concentrations of 6‐OHDA. (G) Quantification analysis of YOD1 in G. (H) Expression of YOD1 in PC12 cells treated with different time of 6‐OHDA. (I) Quantification analysis of YOD1 in H. (J) ICC was used to detect the expression of YOD1 in PC12 cells treated with different concentration of 6‐OHDA. Data represent mean ± SEM; *p < .05; **p < .01; ***p < .001 versus control or 6‐OHDA‐treated control.

Through GEO database analysis, we observed that YOD1 was highly expressed in the substantia nigra of PD patients (Figure 1A,B). To verify the association between YOD1 and PD, we constructed an acute PD model via unilateral striatal 6‐hydroxydopamine (6‐OHDA) injection, a well‐characterized neurotoxin that selectively degenerates dopaminergic neurons.^21^ Western blot analysis demonstrated markedly elevated YOD1 protein levels in the substantia nigra of 6‐OHDA‐lesioned mice versus sham controls (Figure 1C,D). IF co‐staining with neural lineage markers (NeuN for neurons, tyrosine hydroxylase [TH] for dopaminergic neurons, Iba1 for microglia, GFAP for astrocytes) revealed predominant YOD1 expression in dopaminergic neurons, with minimal detection in glial cells (Figure 1E). This neuronal‐specific expression pattern was recapitulated in vitro, where 6‐OHDA treatment (50−100 µM) dose‐dependently and time‐dependently increased YOD1 expression in PC12 cells (Figure 1F−I). IF confirmed these findings, showing nuclear‐to‐cytoplasmic YOD1 redistribution following 6‐OHDA exposure (Figure 1J). To evaluate the effects of other PD‐inducing agents on YOD1 expression, we evaluated the effects of MPP+ on YOD1 expression. And found that MPP+ can also induce up‐regulation of YOD1 expression in a time‐ and dose‐dependent manner (Figure S1). These consistent observations in vivo and in vitro models strongly implicate YOD1 in PD pathogenesis.

### 3.2 YOD1 knockdown attenuates 6‐OHDA‐induced oxidative damage in neuronal cells

**FIGURE 2:**
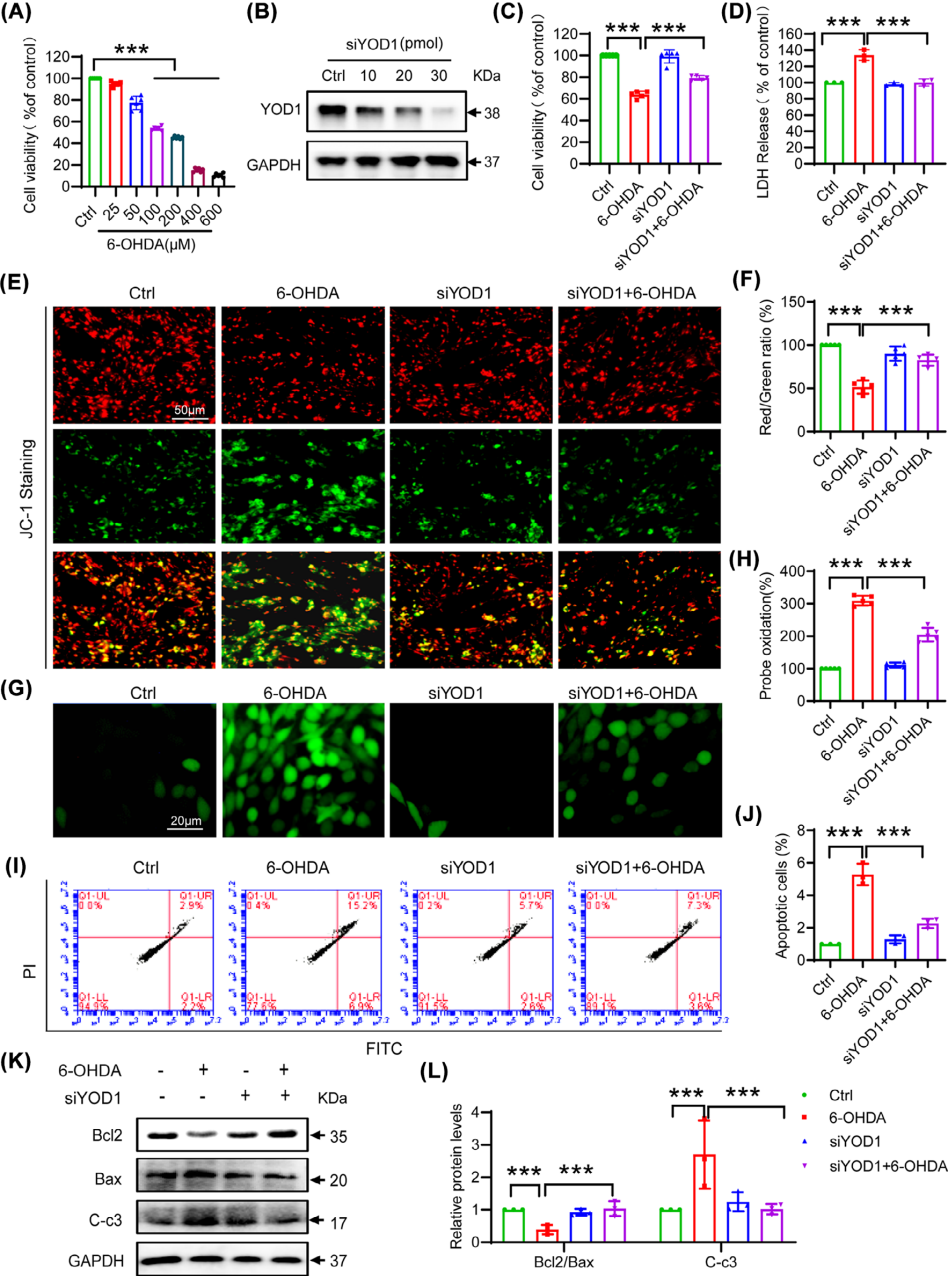
YOD1 knockdown attenuates 6‐OHDA‐induced oxidative stress and apoptosis in PC12 cells. (A) Determination of cell viability after administration of different concentrations of 6‐OHDA(25‐200 µM). (B) Western blot validation of YOD1 knockdown efficiency. (C) YOD1 knockdown improved cell viability induced by 6‐OHDA. (D) YOD1 knockdown reduced LDH levels in cell supernatants after 6‐OHDA stimulation. (E) JC‐1 staining demonstrating that YOD1 knockdown preserves mitochondrial membrane potential. (F) JC‐1 fluorescence quantitative analysis. (G) DCFH‐DA staining showing YOD1 deficiency decreases intracellular ROS accumulation. (H) ROS fluorescence quantitative analysis. (I) Annexin V‐FITC/PI flow cytometry revealing reduced apoptosis in YOD1‐deficient cells. (J) Quantitative analysis of flow cytometry. (K) Western blot analysis of apoptosis‐related proteins (Bax, Bcl‐2 and cleaved caspase‐3) in substantia nigra tissues. (L) Quantitative analysis of western blot results in K by image J. Data represent mean ± SEM of three independent experiments; *p < .05; **p < .01; ***p < .001 versus scrambled control or 6‐OHDA‐treated control.

To investigate the role of YOD1 in neuronal cells, we established an in vitro PD model by treating PC12 cells with 6‐OHDA (0−600 µM, 24 h). MTT assays revealed concentration‐dependent cytotoxicity, with 100 µM 6‐OHDA inducing 40–50% viability reduction (Figure 2A) − this dose was selected for subsequent experiments. Next, we used siRNA to knock down YOD1 in PC12 cells, and western blot results showed that 30 pmol of YOD1 siRNA had the best interference efficiency (Figure 2B). To further explore the effect of YOD1 on neuronal cells, we transfected PC12 cells with 30 pmol of YOD1 siRNA for 24 h and then exposed to 100 µM 6‐OHDA for another 24 h. To confirm the efficacy of YOD1 knockdown in the experimental groups, we performed western blot analysis of YOD1 expression. The results demonstrated successful YOD1 knockdown in PC12 cells, thereby validating that all following results were obtained under conditions of effective YOD1 depletion (Figure S2). Further results showed that YOD1 knockdown significantly attenuated 6‐OHDA‐induced loss of cell viability (Figure 2C) and reduced 6‐OHDA‐induced LDH release (Figure 2D). To further test the effect of YOD1 on oxidative damage, we performed JC‐1 staining and found that YOD1 knockdown inhibited the 6‐OHDA‐induced decrease in mitochondrial membrane potential in PC12 cells (Figure 2E,F). Further ROS staining results showed that knockdown of YOD1 inhibited the 6‐OHDA‐induced intracellular ROS production (Figure 2G,H). The production of intracellular ROS leads to neuronal cell apoptosis, and as we expected, YOD1 knockdown improved 6‐OHDA‐induced PC12 cell apoptosis via flow cytometry (Figure 2I,J). To further verify this result, we used western blot to detect the expression of apoptosis‐related factors Bcl2, Bax and Cleaved‐caspase 3. Results showed that YOD1 knockdown increased the ratio of antiapoptotic switch Bcl2/Bax and inhibited the expression of Cleaved‐caspase3 (Figure 2K,L). The above results indicate that YOD1 knockdown attenuated 6‐OHDA‐induced oxidative stress.

### 3.3 YOD1 deficiency improves motor deficits and dopaminergic neuron degeneration in 6‐OHDA‐induced acute PD model mice

**FIGURE 3:**
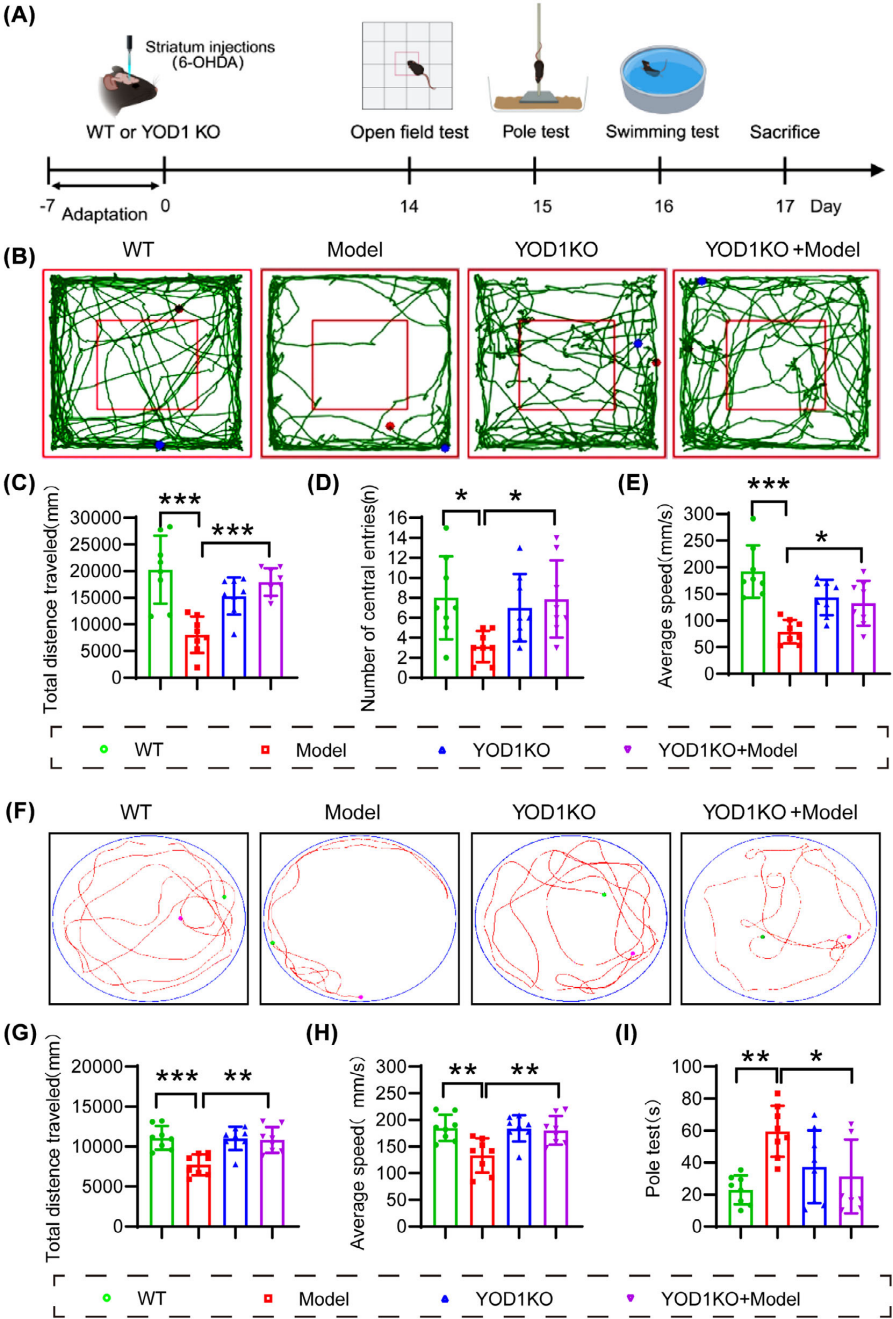
YOD1 knockout significantly improves the motor ability and coordination ability of PD model mice. (A) Experimental timeline schematic. (B) Representative trajectory diagrams of mice in each group in the open field experiment. (C) The total distance travelled by mice in each group in the open field experiment. (D) The number of times mice in each group entered the central area in the open field experiment. (E) The average movement speed of mice in each group in the open field experiment. (F) Representative trajectory diagram of swimming experiment. (G) The total distance travelled by mice in each group during the swimming experiment. (H) The average movement speed of mice in each group during the swimming experiment. (I) Time spent in rod climbing experiment for mice in each group. Data are presented as mean ± SEM (*n* = 10/group); *p < .05; **p < .01; ***p < .001 versus wild‐type controls or Model group.

To further verify the role of YOD1 in PD pathogenesis, 6‐OHDA was injected into the striatum of YOD1 knockout mice to simulate the acute pathological state of PD (Model group). The control group was given an equal amount of solvent. Western blot results confirm YOD1 knockout efficiency (Figure S3). The behavioural changes of WT and model mice were detected 2 weeks later (Figure 3A). The movement trajectories of each group of mice in the OFT (Figure 3B) showed that the movement trajectory, movement distance and time of YOD1 knockout mice in the central area of the open field were significantly higher than those of PD model mice (Figure 3C−E). We further validated our results using the swimming test (Figure 3F). The results showed that the swimming distance and speed of mice in the YOD1 knockout group were higher than those of mice in the model group (Figure 3G,H). The pole climbing experiment also showed that YOD1 knockout significantly improved the motor coordination ability of model mice (Figure 3I).

**FIGURE 4:**
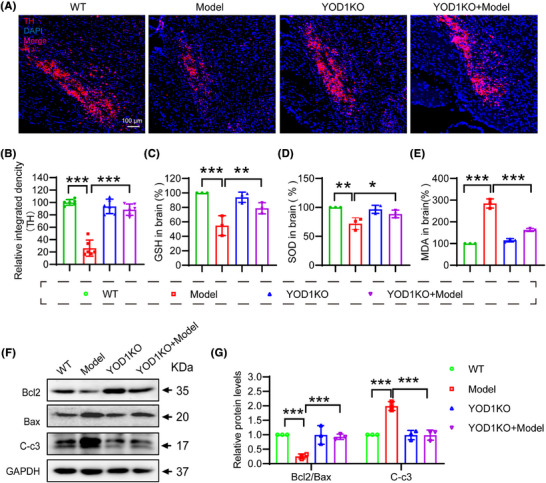
YOD1 deficiency protects against dopaminergic neuron degeneration and oxidative stress in PD model mice. (A) Representative immunofluorescence images of TH‐positive neurons in substantia nigra sections. (B) Quantification of TH+ neuron density (cells/mm^2^). (C−E) Oxidative stress markers in substantia nigra lysates: (C) glutathione (GSH) levels, (D) superoxide dismutase (SOD) activity, and (E) malondialdehyde (MDA) content. (F) Western blot analysis of apoptosis‐related proteins (Bax, Bcl‐2 and cleaved caspase‐3) in substantia nigra tissues. (G) Densitometric quantification of protein expression levels normalized to GAPDH in F. Data represent mean ± SEM; *p < .05; **p < .01; ***p < .001 versus wild‐type controls or Model group.

The selective loss of dopaminergic neurons in the substantia nigra,^22^ characterized by TH depletion, represents a pathological hallmark of PD that disrupts basal ganglia motor circuits.^23^, ^24^ Immunohistochemical analysis revealed that YOD1 knockout mice exhibited an increase in TH+ neuron density compared to PD models (Figure 4A,B), demonstrating significant neuroprotection. Given the established role of oxidative stress in dopaminergic neuron degeneration,^25^ we quantified key redox biomarkers in nigral tissue lysates. The results showed that YOD1 deficiency increased the antioxidant factors GSH and superoxide dismutase (SOD) levels, while decreased the MDA level (Figure 4C−E). Western blot analysis demonstrated that YOD1 deficiency increased the expression of the ratio of Bcl‐2/Bax and reduced cleaved caspase‐3 expression (Figure 4F,G). Suggesting that YOD1 deficiency improves neuronal cell apoptosis in the 6‐OHDA‐induced PD model mice.

### 3.4 YOD1 knockdown confers neuroprotection in chronic A53T model mice

**FIGURE 5:**
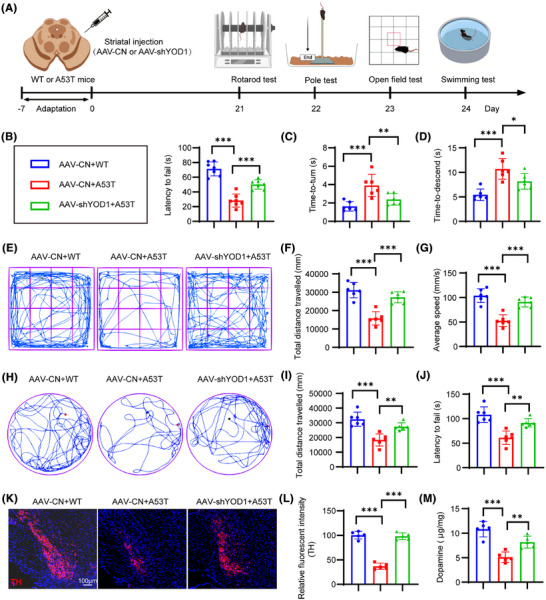
YOD1 knockdown ameliorates motor dysfunction and dopaminergic neuron degeneration in A53T α‐synuclein transgenic mice. (A) Schematic overview of the experimental timeline. (B−D) Motor coordination assessments showing: (B) latency to fall in the rotarod test, (C) turning time and (D) total duration in the pole test. (E−G) Locomotor activity analysis from open field testing: (E) representative movement trajectories, (F) total distance travelled and (G) average velocity. (H−J) Swim test performance: (H) representative swimming paths, (I) total swim distance and (J) average swim speed. (K) Representative immunofluorescence images of tyrosine hydroxylase (TH)‐positive neurons in substantia nigra sections. (L) Quantitative analysis of TH+ neuron density. (M) Dopamine (DA) levels measured in substantia nigra tissue lysates. Data are presented as mean ± SEM; *p < .05; **p < .01; ***p < .001 versus control or 6‐OHDA‐treated control.

To further validate our findings, we generated an A53T α‐synuclein transgenic PD model and delivered AAV‐mediated YOD1 knockdown via stereotaxic striatal injection (Figure 5A). Rotarod test demonstrated that YOD1 knockdown increased fall latency (Figure 5B). Pole test showed that YOD1 knockdown demonstrated faster descent latencies, indicating improved motor coordination (Figure 5C,D). YOD1 knockdown exhibited greater centre zone exploration time and increased total movement distance versus A53T models (Figure 5E−G). Swim test showed that YOD1 knockdown increased mean swim velocity and total swim distance (Figure 5H−J). Similarly, YOD1 knockdown mice exhibited greater TH+ neuron survival (Figure 5K,L) and DA (Figure 5M) content compared to A53T models. Together, these consistent results across acute (6‐OHDA) and chronic (A53T) PD models demonstrate that YOD1 knockdown preserves dopaminergic neurons, maintains DA levels and ameliorates motor deficits through multiple behavioural paradigms, confirming its therapeutic potential for PD intervention.

### 3.5 PKM2 is identified as a potential substrate of YOD1 in PD pathogenesis

**FIGURE 6:**
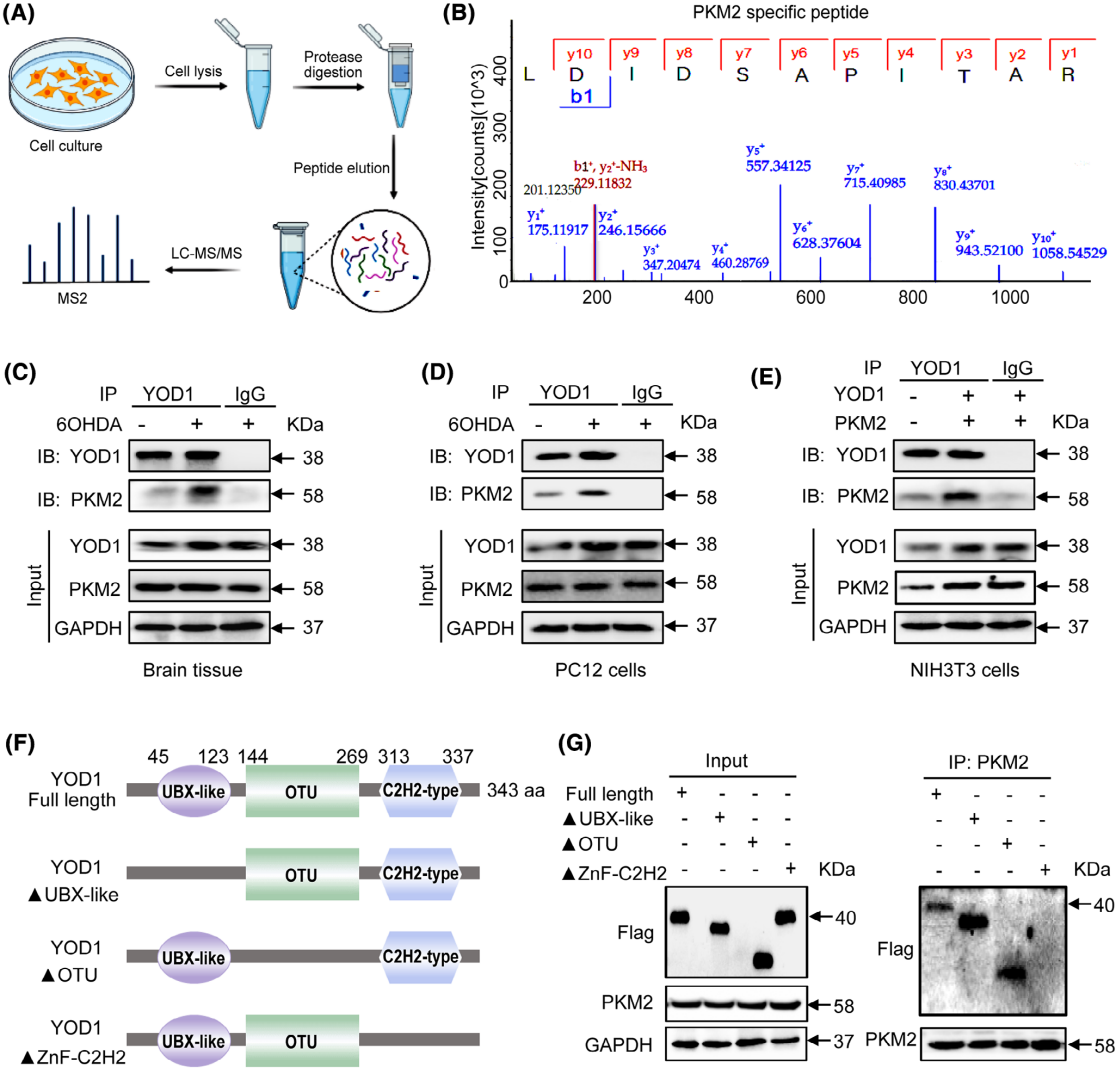
YOD1 directly interacts with PKM2. (A) Experimental workflow for identifying YOD1‐interacting proteins by LC‐MS/MS. (B) MS/MS spectrum from PKM2 peptide. (C) Co‐IP analysis of YOD1 and PKM2 interaction in the substantia nigra of mice infused with or without 100 µM 6‐OHDA. The protein level of PKM2 was detected by western blot. (D) Co‐IP analysis of YOD1 and PKM2 interaction in PC12 cells induced with or without 100 µM 6‐OHDA. (E) Co‐IP analysis of YOD1 and PKM2 interaction in NIH3T3 cells. (F) Schematic diagram of YOD1 structure and design of three different truncated segment plasmids tagged with FLAG (UBX‐like, OTU, Znf‐C2H2). (G) Overexpression of YOD1 and PKM2 by transfer Flag‐YOD1 and His‐PKM2, Flag‐YOD1ΔUBX‐Like and His‐PKM2, Flag‐YOD1ΔOTU and His‐PKM2, Flag‐YOD1ΔZnf‐C2H2 and His‐PKM2 plasmids to NIH3T3 cells. Co‐IP analysis to detect the binding region of YOD1 to PKM2.

To elucidate the molecular mechanism underlying YOD1‐mediated neuroprotection, we performed comprehensive proteomic screening through co‐immunoprecipitation (Co‐IP) coupled with liquid chromatography‐tandem mass spectrometry (LC‐MS/MS). This approach identified PKM2 as a high‐confidence interacting substrate (Figure 6A,B). PKM2 plays an important biological role in PD by regulating glycolysis, oxidative stress and other mechanisms, and is closely related to cellular localization, protein conformation and post‐translational modification of proteins.^26^ Notably, its oligomeric state determines functional specificity − cytoplasmic tetramers exhibit pyruvate kinase activity, while nuclear dimers serve as transcriptional coactivators for Nrf2, the master regulator of antioxidant response elements.^26^, ^27^ Nrf2 orchestrates cellular defence against oxidative stress by activating cytoprotective gene networks that maintain redox balance and prevent apoptosis.^28^, ^29^ Therefore, we speculate that the regulatory effect of YOD1 on oxidative stress may be achieved through binding to PKM2. To verify this conjecture, Co‐IP experiments were performed on mouse brain substantia nigra tissue (Figure 6C), PC12 cells (Figure 6D) and NIH3T3 cells (Figure 6E) that overexpressed both YOD1 and PKM2. In addition, Double fluorescent staining of YOD1 (green) and PKM2 (red) found that YOD1 colocalized with PKM2 (Figure S4). These above results confirmed that PKM2 interacts with YOD1. Domain mapping revealed the C2H2 zinc finger domain (aa 313–337) as critical for binding, as deletion of aa 313–317 (ΔC2H2) completely abolished interaction, while UBX‐like (Δ45‐123) and OTU (Δ144‐269) domain truncations maintained binding capacity (Figure 6F,G). This identifies a novel molecular interface regulating the YOD1‐PKM2 axis in PD pathogenesis.

### 3.6 YOD1 regulates K63‐linked deubiquitination of PKM2

**FIGURE 7:**
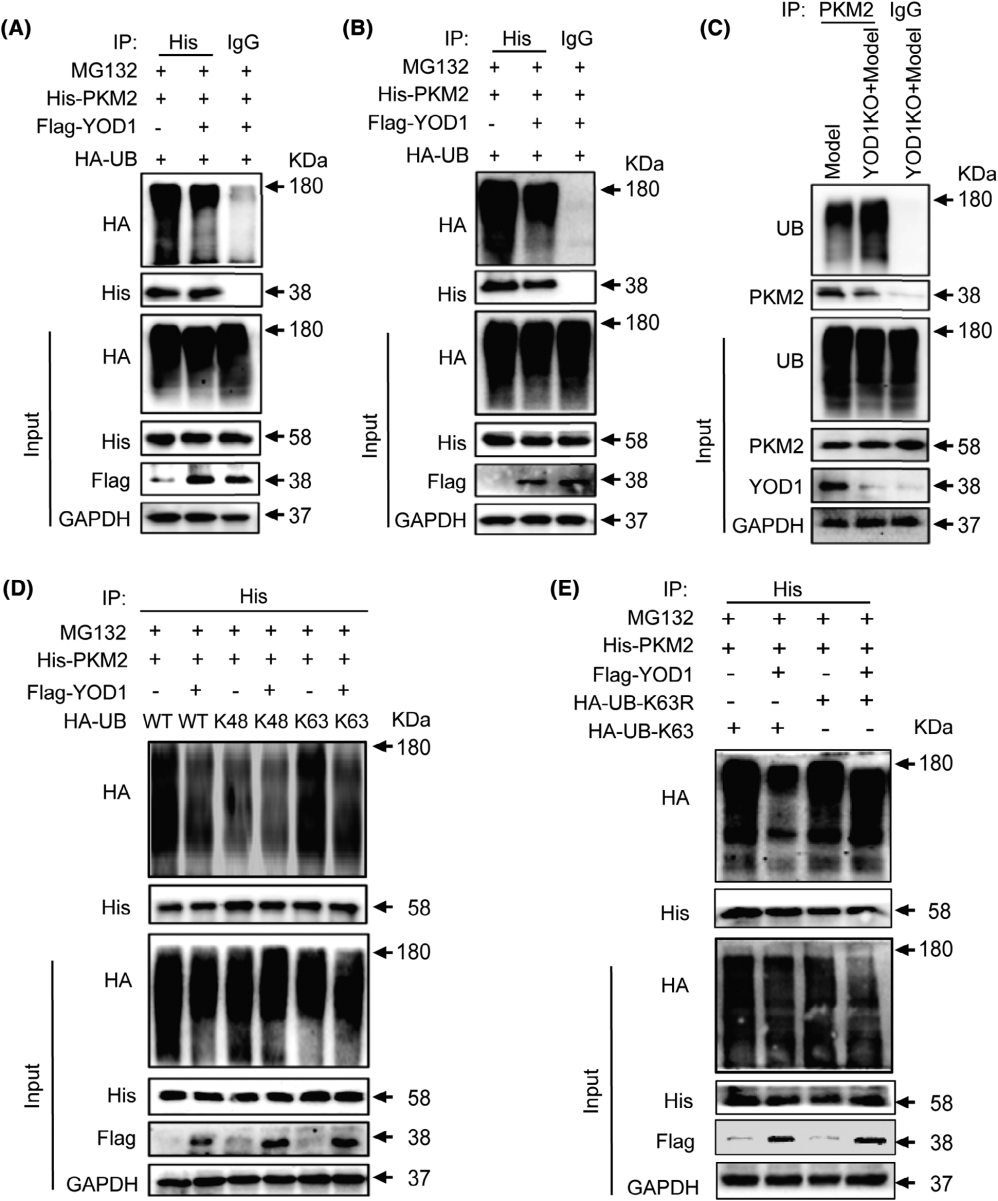
YOD1 regulates PKM2 ubiquitination by removing its K63 ubiquitin chain. (A) Co‐transfection of His‐PKM2, HA‐UB, Flag‐Yod1 or Flag‐CN overexpression plasmids into PC12 cells. PKM2 ubiquitination level was detected by western blot with His‐specific antibodies. (B) Co‐transfection of His‐PKM2, HA‐UB, Flag‐YOD1 or Flag‐CN overexpression plasmids into NIH3T3 cells. PKM2 ubiquitination level was detected by western blot with His‐specific antibodies. (C) Endogenous PKM2 ubiquitination analysis in YOD1 knockout versus wild‐type cells by co‐immunoprecipitation (Co‐IP)/western blot. (D) Co‐transfection of His‐PKM2, HA‐UB, HA‐K48, HA‐K63 and Flag‐YOD1 overexpression plasmids into NIH/3T3 cells. YOD1‐regulated PKM2 ubiquitination pattern was detected by western blot with His‐specific antibodies. (E) Functional validation using K63R ubiquitin mutant confirming the essential role of K63 linkage in YOD1‐mediated PKM2 deubiquitination.

To clarify whether YOD1 can regulate the ubiquitination of PKM2, we co‐transfected HA‐Ub, His‐PKM2 and Flag‐YOD1 or Flag‐CN overexpression plasmids into NIH3T3 cell lines or PC12 cells, and used His magnetic beads to perform Co‐IP experiments, and western blot to detect the ubiquitination of PKM2. Results demonstrated that YOD1 overexpression significantly reduced PKM2 ubiquitination in both PC12 (Figure 7A) and NIH3T3 cells (Figure 7B). Similarly, YOD1 knockout led to a marked increase in PKM2 ubiquitination (Figure 7C). Normally, K48‐linked ubiquitin chains typically target substrates for proteasomal degradation, while K63‐linked chains primarily modulate protein activity,^30^, ^31^ we further characterized the specific ubiquitin linkage affected by YOD1. By co‐expressing Flag‐YOD1, His‐PKM2 and mutant ubiquitin constructs (retaining only K48 or K63 lysine residues) in NIH‐3T3 cells, we observed that the HA‐UbK63 plasmid specifically reduced PKM2 ubiquitination (Figure 7D). This result was further confirmed by the impaired ubiquitination observed with K63R mutant ubiquitin (Figure 7E). Collectively, these findings demonstrate that YOD1 mediates K63‐linked deubiquitination of PKM2, suggesting a regulatory role in modulating PKM2 activity rather than its stability.

### 3.7 YOD1 regulates PKM2 dimerization and Nrf2 signalling pathway to promote oxidative stress in neurons

**FIGURE 8:**
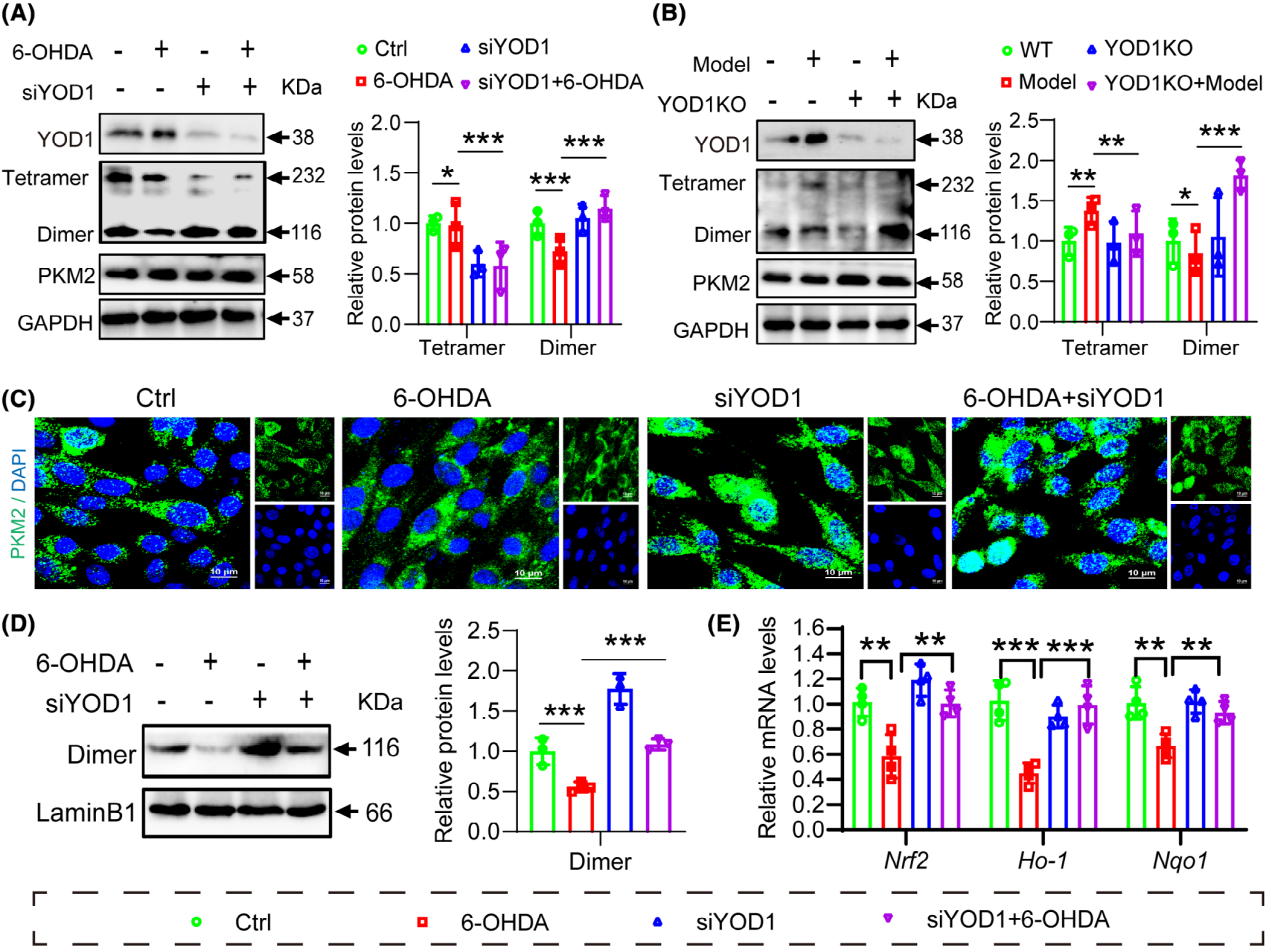
YOD1 modulates PKM2 dimerization and nuclear translocation. (A) PC12 cells were transfected with YOD1 siRNA for 24 h and then stimulated with 100 µM 6‐OHDA for another 24 h. Western blot was used to detect the protein levels of YOD1, PKM2 and YOD1. (B) Comparative assessment of YOD1 and PKM2 protein levels in substantia nigra tissues from wild‐type (WT), 6‐OHDA model (Model), YOD1 knockout (YOD1KO) and YOD1KO+Model groups. (C) PC12 cells were transfected with YOD1 siRNA for 24 h and then stimulated with 100 µM 6‐OHDA for 24 h. Use ICC to detect the nuclear entry of PKM2. (D) PC12 cells were transfected with YOD1 siRNA and stimulated with 100 µM 6‐OHDA for 24 h. Western blot detected the protein expression of Nrf2 in nucleus. (E) RT‐qPCR detection of Nrf2, HO‐1, NQO1 mRNA levels in PC12 cells. YOD1 modulates PKM2 dimerization and nuclear translocation. Data represent mean ± SEM of three independent experiments; *p < .05; **p < .01 versus control siRNA group or 6‐OHDA‐treated control group.

To further elucidate whether the role of YOD1 in oxidative damage involves PKM2 regulation, we performed YOD1 knockdown in PC12 cells and analysed PKM2 expression by western blot. Interestingly, we found that YOD1 knockdown did not affect the protein level and stability of PKM2, but reduced PKM2 tetramer formation while increasing dimeric PKM2 (Figure 8A). This finding was corroborated in brain tissues from YOD1 knockout mice, where PKM2 stability remained unchanged but dimerization was enhanced (Figure 8B). Given that PKM2 dynamically interconverts between tetrameric and dimeric states, with nuclear translocation of dimeric PKM2 activating the Nrf2 antioxidant pathway, we next examined PKM2 subcellular localization. Immunocytochemistry revealed increased nuclear PKM2 accumulation following YOD1 knockout (Figure 8C), a result further validated by fractionation experiments showing elevated nuclear PKM2 levels in YOD1‐deficient PC12 cells (Figure 8D). Since nuclear PKM2 interacts with Nrf2 to induce expression of antioxidant genes HO‐1 and NQO1, we assessed these downstream effectors. Consistent with our hypothesis, YOD1 deficiency upregulated Nrf2 along with its targets HO‐1 and NQO1 by promoting PKM2 nuclear entry (Figure 8E). These results collectively suggest that YOD1 modulates oxidative stress in dopaminergic neurons by regulating PKM2 dimerization and subsequent Nrf2 pathway activation, implicating this mechanism in PD pathogenesis.

## 4 DISCUSSION

In this study, we demonstrate that YOD1 expression is significantly upregulated in both PD model mice and patients with PD. YOD1 deficiency ameliorates motor dysfunction and attenuates oxidative damage in dopaminergic neurons of PD models. Mechanistically, we identify PKM2 as a key substrate of YOD1, showing that YOD1 deficiency mediates K63‐linked deubiquitination of PKM2 to promote its transition from tetramers to dimers. The resulting dimeric PKM2 translocates to the nucleus, where it activates the Nrf2‐mediated antioxidant response pathway by upregulating downstream targets such as HO‐1 and NQO1. This YOD1‐PKM2‐Nrf2 axis critically regulates neuronal oxidative stress and PD‐associated pathology. Our findings establish YOD1 as a promising therapeutic target for mitigating oxidative damage in dopaminergic neurons in PD.

Current research suggests that PD pathogenesis involves multiple interconnected mechanisms, including neuroinflammation, autolysosomal dysfunction, mitochondrial impairment, immune dysregulation, protein misfolding and oxidative stress. These factors mutually reinforce one another, ultimately driving dopaminergic neuron degeneration and disease progression.^32^ Notably, emerging evidence positions oxidative stress as a primary instigator of PD pathology, triggering downstream cascades that exacerbate neuronal damage.^33^ During oxidative stress, PD patients produce a large amount of H2O2 and superoxide anions during the oxidative metabolism of DA, causing abnormal mitochondrial membrane potential in the substantia nigra, resulting in the inability of mitochondria to remove free radicals.^34^, ^35^ This oxidative damage culminates in irreversible neuronal degeneration. Given its central role in PD pathogenesis, targeting key regulators of oxidative stress may represent a promising therapeutic strategy to mitigate dopaminergic neuron loss.

Ubiquitination modification is a highly specific and selective dynamic reversible protein modification process that is widely involved in almost all life activity processes, such as protein degradation, epigenetic regulation and apoptosis.^36^ This modification is to covalently modify the ubiquitin protein to the lysine residue of the substrate protein through the cascade reaction of E1 ubiquitin‐activating enzyme, E2 ubiquitin‐conjugating enzyme and E3 ubiquitin ligase, thereby triggering proteasome degradation.^37^ Conversely, DUBs reverse this process by cleaving the isopeptide bonds between ubiquitin molecules or between ubiquitin and its substrates, thereby providing dynamic regulation of protein fate.^38^ Recent years, more and more studies have found that DUB plays a key role in PD. For example, USP30 can antagonize the PINK1/Parkin‐mediated mitophagy process, triggering excessive accumulation of damaged mitochondria and mediating PD pathology.^39^ OTUD3 plays a key role in improving the pathology of substantia nigra iron deposition in PD by stabilizing iron regulatory protein 2.^40^ Although these studies demonstrate DUB involvement in PD pathology, the specific DUBs regulating oxidative stress − a hallmark of PD − remain largely unidentified. Our current study reveals YOD1 as a novel oxidative stress regulator in PD. In this study, we observed that YOD1 was increased in PD model mice and was mainly expressed in neuronal cells. YOD1 knockout not only attenuated neuronal oxidative damage but also markedly improved motor deficits in PD models. These findings suggest YOD1 as a key mediator of oxidative stress in PD progression and suggest its potential as a therapeutic target.

The biological functions of DUBs are primarily mediated through their substrate specificity. Our study identifies PKM2 as a key substrate of YOD1, demonstrating that YOD1 specifically regulates K63‐linked deubiquitination of PKM2. As a member of the pyruvate kinase family, PKM2 is abundantly expressed in both cancer cells and normal proliferating tissues.^41^ This metabolic enzyme exhibits conformational plasticity, existing in monomeric, dimeric and tetrameric states with distinct biological functions.^42^ The tetrameric form demonstrates high glycolytic activity in the cytoplasm, catalysing the conversion of phosphoenolpyruvate to pyruvate. The dimeric form has low glycolytic activity but can translocate into the nucleus and act as a nuclear transcriptional coactivator regulating gene expression.^43^ Studies have shown that PKM2 is associated with neuronal loss in PD, and upregulation of PKM2 promotes excessive glycolysis and abnormal mitochondrial fusion in neurons, leading to the vulnerability of dopaminergic neurons to 6‐OHDA.^44^, ^45^ Importantly, nuclear PKM2 dimers bind and activate Nrf2, promoting the synthesis of GSH to maintain redox homeostasis in the brain. In the pathological case of PD, aggregated α‐synuclein promotes ROS generation, leading to dopaminergic neuronal death. Conversely, accumulated ROS further facilitates α‐synuclein aggregation, thereby establishing a vicious cycle that exacerbates PD pathogenesis.^26^, ^46^ In addition, the formation of PKM2 and Nrf2 complex can exert antitumour activity. Inhibition of PKM2 activity, resulting in the failure of cytoplasmic retention of Nrf2 to transcribe antioxidant gene expression, induces colon cancer cell oxidative stress.^47^ Emodin, at non‐toxic concentrations, markedly suppresses PKM2 activity and facilitates the dissociation of tetrameric PKM2 into dimers within cells. This dimerization of PKM2 strengthens its interaction with Nrf2, thereby further inducing the activation of the Nrf2/ARE pathway and upregulating a series of cytoprotective genes.^48^ Collectively, the PKM2/Nrf2 axis plays a crucial role in regulating antioxidant capacity. It is suggested that PKM2 may serve as a direct substrate of YOD1 to regulate neuronal oxidative stress. However, we cannot completely exclude potential contributions from other YOD1 substrates in neural cell biology. Importantly, the precise molecular mechanism by which YOD1 promotes PKM2 tetramer‐to‐dimer transition requires further investigation.

In summary, our study elucidates a novel molecular mechanism in PD pathogenesis through the YOD1/PKM2/Nrf2 axis. We demonstrate that YOD1 directly interacts with PKM2 and mediates its K63‐linked deubiquitination, thereby inhibiting the transition from tetrameric to dimeric PKM2. Dimeric PKM2 enters the nucleus to bind and activate the Nrf2 signalling pathway, and promotes the expression of downstream antioxidant factors. These findings not only uncover a previously unrecognized signalling pathway in PD pathology but also identify YOD1 as a potential therapeutic target for mitigating oxidative stress in dopaminergic neurons.

## AUTHOR CONTRIBUTIONS

**Guang Liang**: Supervision, project administration, funding acquisition, conceptualization. **Xia Zhao**: Data curation, writing—original draft, funding acquisition, conceptualization. **Wei Wang**: Project administration, funding acquisition, conceptualization. **Jinfeng Sun**: Software, funding acquisition, data curation. **Fan Chen**: Software, resources, data curation. **Yuqing Zeng**: Data curation, methodology. **Hao Tang**: Data curation, validation. **Luyao Li**: Data curation. **Muzaffar Hammad**: Writing—review and editing. **Xiaoxia Xu**: Methodology, formal analysis. **Ziyao Meng**: Formal analysis. **Qin Yu**: Software. **Linjie Chen**: Software.

## CONFLICT OF INTEREST STATEMENT

The authors declare that they have no competing interests.

## ETHICS STATEMENT

The authors have nothing to report.

## Supporting information



Supporting Information

## Data Availability

The data that support the findings of this study are available on request from the corresponding author. The data are not publicly available due to privacy or ethical restrictions.
