# Supplementary material for: YOD1 regulates oxidative damage of dopamine neurons in Parkinson's disease by deubiquitinating PKM2
Source: Clin Transl Med. 2025 Jul 18;15(7):e70420. doi: 10.1002/ctm2.70420 (PMC12274113; doi:10.1002/ctm2.70420)
Supplement: Supplementary file 1 — Supporting Information [file CTM2-15-e70420-s001.docx]

**YOD1 regulates oxidative damage of dopamine neurons in Parkinson's disease by deubiquitinating PKM2**

Xia Zhao^1,2#*^, Jinfeng Sun^1,3#^, Fan Chen^1,2^, Hao Tang^2^, Yuqing Zeng^2^, Luyao Li^4^, Qin Yu^2^, Linjie Chen^2^, Muzaffar Hammad^2^, Xiaoxia Xu^2^, Ziyao Meng^2^, Guang Liang^1,2*^

*^1^ Department of Pharmacy and Institute of Inflammation, Zhejiang Provincial People's Hospital, Affiliated People's Hospital, Hangzhou Medical College, Hangzhou, Zhejiang, 310014, China*

*^2^ School of Pharmaceutical Sciences, Hangzhou Medical College, Hangzhou, Zhejiang, 311399, China;*

*^3^ Key Laboratory of Natural Medicines of the Changbai Mountain, Ministry of Education, Yanbian University, Yanji, Jilin,133002, P.R. China;*

*^4^ Chemical Biology Research Center, School of Pharmaceutical Sciences, Wenzhou Medical University, Wenzhou, Zhejiang 325035, China*

**Running Title:** YOD1-PKM2 axis mediates oxidative stress in PD

*** To whom correspondence should be addressed:**

Guang Liang, Ph. D, Professor, School of Pharmaceutical Sciences, Hangzhou Medical College, Hangzhou, Zhejiang 311399, China.

1. mail: [wzmcliangguang@163.com](mailto:wzmcliangguang@163.com).

Wei Wang, Professor, Affiliated Yongkang First People’s Hospital, Hangzhou Medical College, Yongkang, Zhejiang 321399, China

E-mail: ykoneway2000@126.com.

Xia Zhao, Ph.D, Professor, School of Pharmaceutical Sciences, Hangzhou Medical College, Hangzhou, Zhejiang 311399, China

E-mail: xiazhao@hmc.edu.cn.

**^#^** These authors contribute to this study equally.

***Supplemental Tables***

**Table S1. Antibodies used in this study**

| **Antibodies** | **Cat. NO** | **Source** | **Dilution** |
| --- | --- | --- | --- |
| YOD1 | ABS134899 | Absin | WB:1:1000/IF:1:200 |
| PKM2 | 15822-1-AP | Proteintech | WB:1:1000 |
| P-PKM2 (Ser37) | DF7772 | Affinity | WB:1:1000/IF:1:200 |
| BAX | 2774 | CST^a)^ | WB:1:1000 |
| BCL2 | 15071 | CST | WB:1:1000 |
| Cleaved caspase3 | 9661 | CST | WB:1:1000 |
| Nrf2 | 12127s | CST | WB:1:1000 |
| TH | 2792 | CST | IF:1:1000 |
| Lamin B1 | 66095 | Proteintech | IF:1:1000 |
| NeuN | 24307s | CST | IF:1:200 |
| GAPDH | AF0006 | Beyotime | WB:1:2000 |
| His-tag | 2365s | CST | WB:1:1000 |
| HA-tag | 3724s | CST | WB:1:1000 |
| Flag-tag | 20543-1-AP | Proteintech | WB:1:500 |
| UB | 43124 | CST | WB:1:1000 |
| Anti-rabbit IgG HRP | A0208 | Beyotime | WB:1:2000 |
| Anti-Mouse IgG HRP | A0216 | Beyotime | WB:1:2000 |
| Alexa Fluor® 594 | 8889 | CST | IF: 1:500 |
| Alexa Fluor® 488 | 4412 | CST | IF: 1:500 |

1. Cell Signaling Technology

**Table S2. The information of reagents or resource.**

| **Primer sequences used in this study** |
| --- |

| *Ho-1* | Forward | 5’-AGGTCCTGAAGAAGATTGC-3’ |
| --- | --- | --- |
|  | Reverse | 5’-TCTCCAGAGTGTTCATTCG-3’ |
| *Nqo1* | Forward | 5’- CGAATCTGACCTCTATGCTAT -3’ |
|  | Reverse | 5’- GCGTCCTTCCTTATATGCTA -3’ |
| *Yod1* | Forward | 5’-GGAAGGAGGAGTCTTGAATC-3’ |
|  | Reverse | 5’- CGTAGTGAATGCCATCGTA-3’ |
| *Nrf2* | Forward | 5’-TACGCAGGAGAGGTAAGAA-3’ |
|  | Reverse | 5’- AAGGAACACATTGCCATCT -3’ |
| *Yod1-KO* | Forward | 5’-ACCAATTTTTCGTTTTCCCTGTGT-3’ |
|  | Reverse | 5’-CTCCACAAGGCTTTCCACATTAC-3’ |
| *Gapdh* | Forward | 5’-TGCCCAGAACATCATCCCT-3’ |
|  | Reverse | 5’-GGTCCTCAGTGTAGCCCAAG-3’ |
| \| **siRNA used in this study** \| \| --- \| | | |
| Yod-1-Mus | Sense | 5’-GGGCAAUCGAGAUCUCAAUTT-3’ |
|  | Antisense | 5’-AUUGAGAUCUCGAUUGCCCTT-3’ |

***Supplemental Figures***


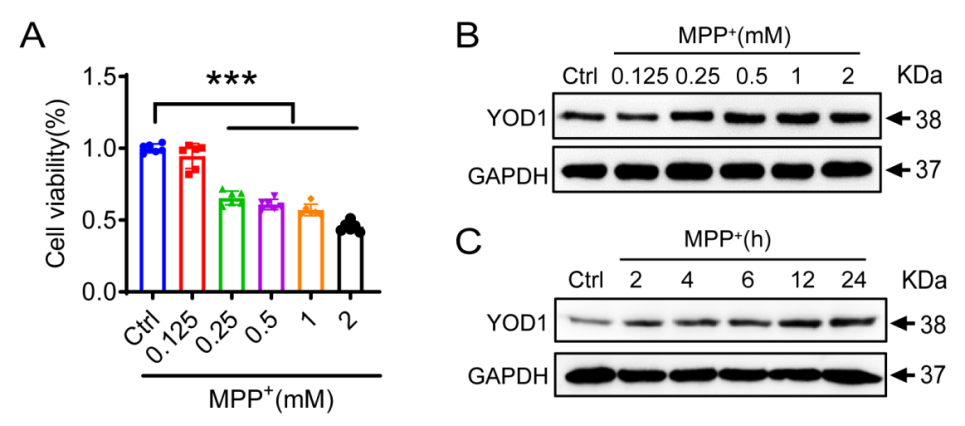


**Figure S1. MPP+ stimulated YOD1 expression in dose- and time- dependent manner.** (A) PC12 cells were treated with different doses of MPP⁺ (0、0.125、0.25、0.5、1、2mM) for 24 hours, and cell viability was assessed using the MTT assay.(B) YOD1 protein expression was analyzed by Western blotting following 24-hour exposure to the indicated concentrations of MPP⁺ (0、0.125、0.25、0.5、1、2mM). (C) Time-course (2、4、6、12、24h) analysis of YOD1 expression after treatment with 0.25 mM MPP⁺. Data are representative of three independent experiments. Data are mean ± SEM; *p < 0.05, **p < 0.01 versus control.


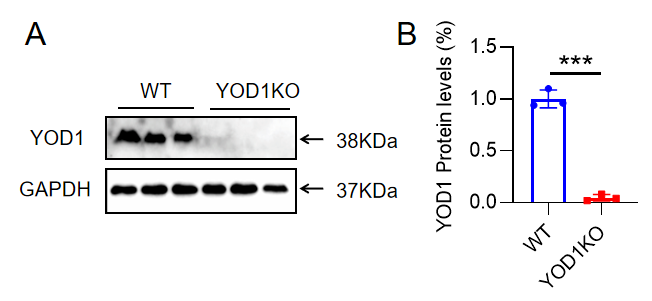


**Figure S2** Knockout efficiency of YOD1 mice. (A) YOD1 protein expression levels in brain tissue of wild-type (WT) and YOD1^–/–^ (KO) mice(n= 3 samples/Group). (B) Quantitative graph of YOD1 expression in panel B. Data are mean ± SEM; ***p<0.001 versus WT group.


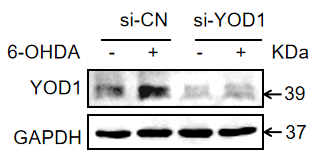


**Figure S3** Knockout efficiency of YOD1 in PC12 cells. PC12 cells were transfected with either control siRNA (si-CN) or YOD1-targeting siRNA (si-YOD1) for 24 h, followed by treatment with 6-OHDA for an additional 24 h. YOD1 protein expression was then assessed by Western blotting. Data are representative of three independent experiments.


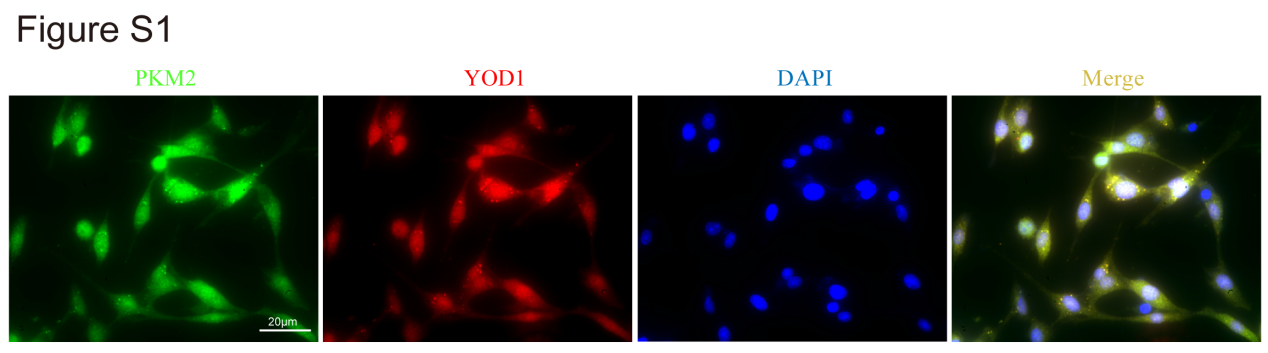


**Figure S4. YOD1 co-localized with PKM2 in PC12 cells.** IF staining of YOD1 (green) and PKM2 (Red) in PC12 cells .
